# Artificial Intelligence in East African Agriculture: A Systematic Review of Applications, Adoption, and Implications for Smallholder Farmers

**DOI:** 10.1155/tswj/5128133

**Published:** 2026-06-18

**Authors:** Zebenay Shitaye, Manale Andargie Embyale, Tewodros Adane Nega, Getachew Eshetu Gidelew, Marta Mohammed

**Affiliations:** ^1^ Department of Rural Development and Agricultural Extension, College of Agriculture and Natural Resource, Debere Markos University, Debere Markos, Ethiopia, dmu.edu.et; ^2^ Department of agricultural Economics, College of Agriculture and Natural Resource, Debere Markos University, Debere Markos, Ethiopia, dmu.edu.et; ^3^ Department of Agricultural Economics, College of Agriculture and Environmental Science, Debark University, Debark, Ethiopia, dku.edu.et; ^4^ Department of Rural Development and Agricultural Extension, College of Agriculture, Wollo University, Dessie, Ethiopia, wu.edu.et

**Keywords:** adoption, agricultural technology, artificial intelligence, climate-smart agriculture, digital agriculture, East Africa, precision agriculture, smallholder farmers

## Abstract

Artificial intelligence (AI) has evolved over several decades and is increasingly recognized as a transformative tool for improving agricultural productivity, resilience, and access to information, particularly in smallholder farming systems such as those in East Africa. This systematic literature review synthesizes existing evidence on the applications, adoption dynamics, implications, and policy considerations of AI in East African agriculture over the period 1985–2025. The study follows PRISMA guidelines and draws on peer‐reviewed articles, conference papers, and institutional reports retrieved from major academic databases, including Scopus, Web of Science, and Google Scholar. A thematic analysis approach was used to organize and interpret the findings. The review shows that early developments in AI‐related agricultural technologies were limited and largely experimental, but advancements in digital technologies, mobile connectivity, remote sensing, and data analytics have significantly expanded AI applications in recent years. Key application areas identified include AI‐powered advisory services, precision agriculture, crop and pest monitoring, financial and market intelligence, and climate‐smart agriculture. These technologies support farmers by enabling real‐time, data‐driven decision‐making, improving resource use efficiency, and enhancing access to agricultural information and markets. Despite these advancements, the adoption of AI among smallholder farmers in East Africa remains relatively low and uneven. The review identifies several factors influencing adoption, including education, digital literacy, access to extension services, infrastructure availability, income levels, and institutional support. Major barriers include limited rural infrastructure, high costs, inadequate digital skills, weak integration with extension systems, and data‐related constraints. Although AI offers promising benefits in terms of productivity, information access, and inclusivity, concerns remain regarding digital inequality, affordability, data privacy, and potential exclusion of marginalized groups. From a policy perspective, the study underscores the importance of strengthening digital infrastructure, investing in capacity building, enhancing extension services, and promoting inclusive public–private partnerships to support the effective deployment of AI technologies. Overall, the review concludes that although AI has significant potential to transform East African agriculture, its impact depends on addressing systemic constraints and ensuring that technologies are accessible, affordable, and aligned with the needs of smallholder farmers. The study also identifies research gaps and suggests future directions for advancing AI integration in the region.

## 1. Introduction

### 1.1. Background and Significance

Agriculture is at the heart of economic and social life in East Africa. It serves as a major source of income, employment, and food for millions of people, particularly in rural areas. In countries such as Kenya, Ethiopia, Uganda, Tanzania, and Rwanda, agriculture contributes a substantial share of GDP and employs a large proportion of the population [1]. Smallholder farmers dominate the sector, producing a significant share of the region′s food despite operating on small plots of land and with limited resources [2].

However, agricultural productivity in the region remains low. This is largely due to a combination of environmental, economic, and institutional challenges. Climate change has increased the variability of rainfall and the frequency of extreme weather events such as droughts and floods, which negatively affect crop production [3]. Soil degradation, limited access to improved inputs, and inadequate extension services further constrain agricultural performance [2]. Moreover, farmers often lack access to timely and accurate information on weather conditions, pest outbreaks, and market prices, making it difficult to make informed decisions [4].

In this context, digital technologies are increasingly recognized as important tools for agricultural transformation. Among these, artificial intelligence (AI) stands out due to its ability to process large volumes of data, identify patterns, and provide actionable insights. For this review, we define AI as a family of technologies including machine learning (ML), deep learning (DL), computer vision (CV), natural language processing (NLP), and predictive analytics that enable machines to perform tasks that normally require human intelligence. This definition excludes basic digital services (e.g., simple SMS weather alerts) that do not involve learning or pattern recognition. AI applications in agriculture include ML models for yield prediction, CV for pest and disease identification, remote sensing for crop monitoring, and digital advisory services delivered via mobile phones [5, 6].

Globally, AI has already begun to transform agricultural systems, particularly in developed countries. In East Africa, however, its adoption is still at an early stage. Nevertheless, the region presents unique opportunities for AI integration. The rapid expansion of mobile phone usage and digital platforms provides a strong foundation for delivering AI‐enabled services to farmers [7]. At the same time, challenges such as limited infrastructure, low digital literacy, and affordability constraints continue to hinder widespread adoption.

### 1.2. Rationale for the Review

Despite the growing interest in AI and digital agriculture, there is still a lack of comprehensive studies that focus specifically on East Africa. Much of the existing literature tends to examine individual technologies or specific case studies, without providing a broader understanding of how AI is being applied across the agricultural sector in the region. To contextualize our findings, we compare East Africa with other smallholder farming regions South Asia (India, Bangladesh), West Africa (Nigeria, Ghana), and Latin America (Colombia, Brazil) highlighting shared challenges and unique pathways.

Understanding the factors that influence adoption is particularly important. The success of AI technologies depends not only on their technical capabilities but also on their accessibility, affordability, and relevance to smallholder farmers. Factors such as education, access to information, institutional support, and socioeconomic conditions play a key role in shaping adoption decisions [8].

In addition, the use of AI in agriculture raises important policy and ethical considerations. Issues such as data ownership, privacy, inclusiveness, and the potential for widening digital inequalities must be carefully addressed. Without deliberate efforts to ensure equitable access, there is a risk that the benefits of AI may not reach the most vulnerable farmers.

Therefore, this review aims to provide a clear and comprehensive synthesis of AI in East African agriculture. Specifically, it seeks:

• To explore the main ways AI is currently being used in East African agriculture, including advisory services, precision farming, crop and pest monitoring, financial and market systems, and climate‐smart agriculture
• To understand how AI technologies are being adopted by smallholder farmers across East Africa and the patterns that characterize this adoption
• To identify the key factors that influence farmers′ decisions to adopt AI technologies, including socioeconomic conditions, access to infrastructure, institutional support, and digital readiness
• To examine the main challenges and barriers that limit the effective adoption and use of AI technologies in smallholder farming systems
• To assess the practical implications of AI for smallholder farmers, particularly in terms of productivity, access to information, inclusivity, and potential risks
• To review the policy and development measures needed to support wider adoption and effective scaling of AI technologies in the region
• To highlight existing research gaps and suggest future directions for research on AI in East African agriculture

By bringing together existing evidence in a systematic and accessible manner, this review contributes to a better understanding of how AI can support inclusive and sustainable agricultural development in East Africa.

## 2. Methodology

This study employed a systematic literature review (SLR) approach to examine how AI is being applied in agriculture across East Africa, with particular emphasis on smallholder farming systems. The review followed the Preferred Reporting Items for Systematic Reviews and Meta‐Analyses (PRISMA) guidelines to ensure that the process of identifying, screening, selecting, and analyzing studies was transparent, systematic, and reliable.

### 2.1. Search Strategy and Sources of Literature

Relevant studies were collected from major academic databases, including Scopus, Web of Science, and Google Scholar. In addition to peer‐reviewed journal articles, conference papers, institutional reports, technical documents, and policy publications were also reviewed to capture practical experiences and recent developments that may not yet be fully represented in journal publications.

The review covered studies published between 1985 and 2025. The review period also captures the evolution from basic digital agricultural tools to more advanced AI‐driven technologies such as ML, predictive analytics, remote sensing, and precision agriculture.

The literature search was conducted between January and March 2026. To identify relevant studies, different keywords and Boolean operators were carefully combined. The main search terms included “artificial intelligence,” “machine learning,” “deep learning,” “digital agriculture,” “precision agriculture,” “smart farming,” “smallholder farmers,” “East Africa,” and “Sub‐Saharan Africa.”

Country‐specific keywords such as Ethiopia, Kenya, Uganda, Tanzania, and Rwanda were also included to improve the relevance of the search results. An example of the search string used in the databases was (“artificial intelligence” OR “machine learning” OR “deep learning”) AND (“digital agriculture” OR “precision agriculture” OR “smart farming”) AND (“smallholder farmers”) AND (“East Africa” OR “Sub‐Saharan Africa”).

### 2.2. Screening and Study Selection Process

The study selection process was conducted in several stages following the PRISMA framework.

1. First, all retrieved records were exported into a reference management system, and duplicate studies were removed.
2. Second, the titles and abstracts of the remaining studies were screened to assess their relevance to the objectives of the review.
3. Third, studies that appeared relevant during the initial screening were subjected to full‐text review.
4. Finally, the full articles were carefully evaluated to determine whether they met the inclusion criteria of the study.

To improve the reliability of the review process and reduce selection bias, two reviewers independently screened and assessed the articles. Any disagreements regarding study inclusion or exclusion were resolved through discussion and mutual agreement.

### 2.3. Inclusion and Exclusion Criteria

Studies were included in the review if they focused on the application of AI or AI‐related technologies in agriculture; were conducted in East Africa or the wider sub‐Saharan African region, addressed issues related to smallholder farmers or rural agricultural systems; provided empirical findings, practical applications, field evidence, or case studies and were published in English between 1985 and 2025.

Studies were excluded if they focused on nonagricultural applications of AI such as healthcare, banking, or manufacturing; were purely theoretical or conceptual without practical agricultural application; focused mainly on large‐scale commercial farming systems with little relevance to smallholder agriculture; were not related to the African context; were editorials, opinion papers, duplicated records, incomplete studies, or lacked sufficient methodological information.

For example, studies discussing AI in medical diagnosis, industrial robotics, or financial systems were excluded during the screening process. Similarly, papers related to agriculture but without any AI or digital technology component were also removed.

### 2.4. Data Extraction and Analysis

After the final selection of studies, important information was systematically extracted using a structured data extraction framework. The extracted information included author(s) and year of publication, country or study area, type of AI technology used, agricultural sector addressed, research methods applied, major findings, adoption factors, impacts on productivity and livelihoods, and key implementation challenges.

The collected information was analyzed using thematic analysis. This approach helped identify recurring patterns, major trends, and common challenges across the reviewed studies. The findings were grouped into four major themes: Types of AI applications used in agriculture, factors affecting AI adoption among smallholder farmers, impacts of AI on productivity and rural livelihoods, and challenges limiting implementation and sustainability.

### 2.5. Reliability and Limitations of the Review

This review aimed to provide a comprehensive and up‐to‐date understanding of how AI is shaping agriculture in East Africa. However, some limitations should be acknowledged. The review relied mainly on published English‐language sources, which means that some unpublished studies, local project reports, or studies published in other languages may not have been captured. In addition, some ongoing innovations in AI‐based agriculture may still be undocumented in academic databases.

**Figure 1:**
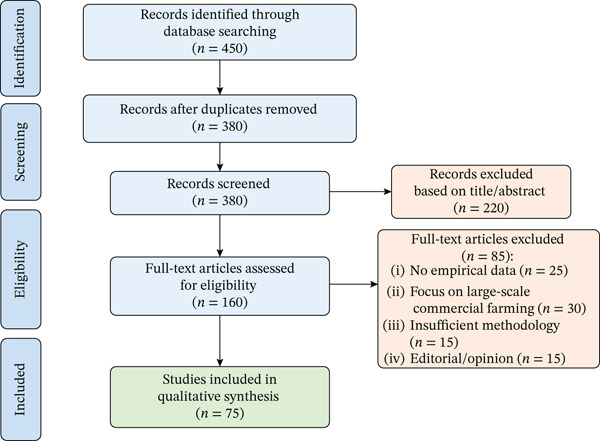
The PRISMA flow diagram summarizes the study selection process.

Despite these limitations, the use of a systematic review approach and adherence to PRISMA guidelines strengthened the transparency, consistency, and credibility of the study. The PRISMA flow diagram (Figure 1) summarizes the process of study identification, screening, eligibility assessment, exclusions, and final inclusion. Care was taken to ensure that the numerical counts presented in the diagram are logically consistent and clearly reconciled across all stages of the review process.

## 3. Detailed Review Literature

### 3.1. AI Applications in East African Agriculture

#### 3.1.1. AI‐Powered Advisory Services

AI‐powered advisory services have become an important innovation in East African agriculture, especially for smallholder farmers who often face constraints in accessing timely and reliable extension services. These systems utilize ML, big data, remote sensing, and climate analytics to provide real‐time, location‐specific recommendations on crop management, pest and disease control, soil fertility, irrigation, and weather forecasting. By integrating multiple data sources such as historical climate data, soil maps, and satellite imagery, AI tools can generate tailored guidance that improves farm‐level decision‐making and productivity. This is particularly relevant in East Africa, where agriculture is highly dependent on rainfall and vulnerable to climate variability [9, 10].

Most AI advisory platforms are delivered through mobile phones using applications, SMS‐based services, or conversational chatbots, making them relatively accessible even in rural settings where infrastructure may be limited. These platforms often combine NLP with agronomic knowledge bases to interpret farmers′ questions and respond in local languages or simplified formats. AI‐powered virtual assistants and chatbots act as digital extension agents, allowing farmers to ask questions about planting dates, fertilizer use, or pest outbreaks and receive immediate responses. In East Africa, digital extension initiatives have increasingly complemented traditional extension systems by extending outreach to remote farmers and reducing the information gap between research institutions and farming communities [11].

In addition to advisory chatbots, AI systems increasingly incorporate geospatial technologies and remote sensing to monitor crop conditions and environmental risks. Satellite imagery, drones, and IoT‐based sensors can be analyzed using AI algorithms to detect early signs of crop stress, pest infestation, water shortages, or nutrient deficiencies. This allows for early warning systems that help farmers take preventive actions before significant losses occur. AI‐based weather forecasting tools also provide localized predictions that assist farmers in planning planting, irrigation, and harvesting activities. Such climate‐smart advisory services are particularly valuable in East Africa, where climate change has increased the frequency of droughts and erratic rainfall patterns, directly affecting agricultural output [12, 13].

Generative AI is further transforming advisory services by enabling more interactive and adaptive knowledge delivery systems. Tools such as FarmerChat and similar generative AI‐based platforms use large language models to synthesize agricultural knowledge and provide context‐aware responses tailored to individual farmer queries. Unlike traditional rule‐based systems, generative AI can learn from diverse datasets and generate explanations, recommendations, and even problem‐solving guidance in conversational form. This enhances user engagement and makes agricultural knowledge more accessible, especially for farmers with limited formal education. These systems can also integrate local data inputs, making their recommendations more relevant to specific farming contexts and conditions [13, 14].

Despite the promising benefits, the adoption and effectiveness of AI‐powered advisory services in East Africa face several challenges. Limited internet connectivity, low digital literacy, language diversity, and inadequate access to smartphones can restrict the reach of these technologies among rural farming communities. In addition, the accuracy and reliability of AI‐generated recommendations depend heavily on the quality and availability of local data, which is often limited in many regions. Institutional barriers, such as weak integration between digital platforms and public extension systems, also hinder scalability. Addressing these challenges requires investments in digital infrastructure, capacity building for farmers and extension agents, development of localized content, and stronger collaboration between governments, private sector actors, and development partners to ensure inclusive and sustainable implementation of AI‐based advisory systems [10, 15].

#### 3.1.2. Precision Agriculture

Precision agriculture, supported by AI, represents a shift from traditional uniform farming practices to more data‐driven and site‐specific farm management. In East Africa, where smallholder farmers dominate and often face challenges such as limited access to inputs, climate variability, and declining soil productivity, precision agriculture provides an opportunity to improve efficiency and resilience. AI integrates data from diverse sources such as satellite imagery, drones, IoT sensors, and weather stations to generate actionable insights tailored to specific farm conditions. These technologies enable farmers to make more informed decisions on planting, input use, and farm management, ultimately improving productivity while reducing waste and environmental pressure [5, 9, 16, 17].

Soil health monitoring is a key component of precision agriculture and plays an essential role in maintaining long‐term agricultural productivity. AI‐powered systems analyze data from soil sensors, remote sensing platforms, and laboratory soil tests to assess important soil characteristics such as moisture levels, nutrient availability, salinity, and pH. ML algorithms help identify variability within and across fields, allowing for the creation of detailed soil maps that guide farmers in applying inputs more precisely. This enables practices such as variable‐rate application of fertilizers and soil amendments, ensuring that each part of the field receives the appropriate treatment. Such approaches reduce input costs, minimize environmental degradation, and help restore soil fertility, which is particularly important in regions of East Africa where soil erosion and nutrient depletion are persistent problems [6, 18, 19].

AI also contributes significantly to irrigation management by enabling farmers to use water more efficiently through smart and data‐driven systems. IoT‐based soil moisture sensors, combined with AI algorithms and weather forecasting models, allow continuous monitoring of crop water needs and environmental conditions. These systems can recommend the optimal timing and amount of irrigation, helping to avoid both water stress and overirrigation. In water‐scarce and rain‐fed farming systems common in East Africa, such technologies are particularly valuable for improving water‐use efficiency and reducing vulnerability to drought. In addition, AI‐based irrigation systems can integrate climate forecasts and historical data to support adaptive irrigation planning, thereby enhancing climate resilience and supporting sustainable water resource management [5, 13, 20, 21].

Fertilizer optimization is another important area where AI‐driven precision agriculture adds value. In many smallholder farming systems, fertilizers are often applied uniformly without considering the specific nutrient needs of different sections of a field, leading to inefficiencies and environmental harm. AI‐based tools analyze soil test results, crop types, growth stages, and environmental variables to generate precise fertilizer recommendations. ML models can predict nutrient requirements and support variable‐rate fertilizer application, ensuring that nutrients are applied where and when they are needed most. This not only improves crop yields and reduces input costs but also minimizes negative environmental impacts such as nutrient leaching, soil acidification, and greenhouse gas emissions. As a result, AI‐enabled fertilizer management supports both productivity and sustainability goals in agriculture [9, 14, 16, 22].

Overall, precision agriculture powered by AI offers a practical and promising approach to improving agricultural productivity, sustainability, and resilience in East Africa. By enabling farmers to make decisions based on real‐time and localized data, these technologies help optimize resource use and reduce uncertainties associated with farming. However, despite its potential, adoption remains limited due to challenges such as high initial investment costs, limited access to digital infrastructure, insufficient technical skills among farmers, and fragmented landholding systems. There is also a need for stronger institutional support, better integration with extension services, and increased awareness among farmers about the benefits of these technologies. Addressing these challenges through investments in infrastructure, capacity building, affordable digital tools, and supportive policies will be crucial for scaling precision agriculture and ensuring that its benefits reach smallholder farmers across East Africa [9, 10, 17].

#### 3.1.3. Crop and Pest Monitoring

Crop and pest monitoring has been greatly improved through the application of AI, particularly using CV and ML techniques. These technologies allow farmers and agricultural stakeholders to detect plant diseases, pest infestations, and crop stress at early stages by analyzing images captured using smartphones, drones, or field‐based cameras. Instead of relying solely on manual inspection which can be time‐consuming, subjective, and dependent on limited extension support AI‐based systems provide rapid and often real‐time diagnostics. This is especially important in East Africa, where smallholder farmers frequently face delays in accessing expert advice. By enabling early detection and timely response, AI tools help reduce crop losses, improve yields, and enhance overall farm productivity [9, 13, 16, 23].

At the technical level, crop monitoring systems commonly use DL models such as convolutional neural networks (CNNs), as well as more recent architectures like vision transformers (ViTs) and object detection frameworks such as YOLO. These models are trained on large datasets of labeled crop images to recognize patterns associated with specific diseases or pest damage. Farmers can upload images through mobile applications or web platforms, and the AI system processes the input to generate a diagnosis along with suggested management practices, such as pesticide application, crop rotation, or cultural control methods. Recent advancements in DL have improved classification accuracy and robustness under varying field conditions, making these systems increasingly practical for real‐world agricultural use [23–25].

The use of drones (unmanned aerial vehicles) and satellite imagery further strengthens crop and pest monitoring by enabling large‐scale surveillance of agricultural fields. Drones equipped with multispectral or thermal cameras can capture high‐resolution images that reveal subtle variations in crop health, which are then analyzed using AI algorithms to identify affected areas. This approach allows for the mapping of pest infestations and disease spread across entire fields, supporting targeted interventions such as localized spraying of pesticides rather than blanket application. Such precision reduces chemical use, lowers costs, and minimizes environmental impacts. In addition, remote sensing combined with AI can monitor vegetation indices (such as NDVI) to assess crop vigor and detect anomalies early [5, 13, 18].

AI‐based crop and pest monitoring systems also increasingly incorporate predictive analytics to provide early warning services. By combining historical datasets, weather information, and real‐time observations, ML models can forecast the likelihood of pest outbreaks or disease epidemics under specific environmental conditions. These predictive capabilities enable farmers to adopt preventive strategies rather than reactive measures, thereby reducing the severity of potential damage. Additionally, AI‐powered mobile advisory platforms and chatbots can communicate alerts and recommendations in simple language, making them accessible even to farmers with limited formal education. This kind of digital support helps bridge the gap created by weak extension systems and limited access to agronomic expertise in rural areas [12, 14, 22].

Despite the clear benefits, several challenges hinder the widespread adoption of AI‐based crop and pest monitoring systems in East Africa. These include limited access to smartphones and reliable internet connectivity, lack of high‐quality and locally relevant training datasets, and low levels of digital literacy among farmers. In addition, AI models developed in other regions may not always generalize well to local crops, pests, and environmental conditions, which can affect their accuracy. There are also institutional and infrastructural constraints, such as weak integration between digital platforms and agricultural extension services. Overcoming these challenges will require investments in rural digital infrastructure, development of locally trained AI models, farmer training programs, and stronger collaboration between governments, research institutions, and private sector actors to ensure that these technologies are accessible, reliable, and beneficial to smallholder farmers [5, 13].

#### 3.1.4. Financial and Market Intelligence

In East Africa, where most farmers are smallholders with limited access to formal financial services, AI is gradually transforming how financial and market systems operate. One of the longstanding challenges in the region is that many farmers cannot access credit from banks due to a lack of collateral, formal credit history, or reliable financial records. AI is helping to address this gap by enabling alternative credit assessment methods. ML models can analyze nontraditional data sources such as mobile phone usage patterns, mobile money transactions, farm production records, and even satellite data to estimate a farmer′s creditworthiness. In countries like Kenya, where digital financial ecosystems are relatively well developed, fintech platforms have started using AI‐driven credit scoring to extend loans to farmers who would otherwise be excluded from formal banking systems. This has contributed to improved access to finance, allowing farmers to invest in inputs such as improved seeds, fertilizers, and irrigation technologies, which can ultimately enhance productivity and livelihoods [10, 26, 27].

In addition to credit scoring, AI‐powered financial platforms in East Africa are increasingly integrated with mobile money services, which are widely adopted across rural and urban populations. These platforms often provide bundled services that include input financing, digital payments, savings, and sometimes insurance products. AI algorithms help tailor these services to individual farmers by analyzing factors such as farm size, crop type, seasonal cycles, and repayment behavior. For instance, loan repayment schedules can be aligned with harvest periods to reduce financial pressure on farmers. In Kenya, the integration of digital financial services with agricultural advisory platforms has enabled farmers to access not only credit but also relevant agronomic advice and input recommendations through a single digital channel. This kind of integration reduces transaction costs, improves convenience, and strengthens the overall efficiency of service delivery in rural areas [9, 13, 28, 29].

AI is also playing an important role in improving access to market information, which is a critical constraint for many farmers in East Africa. Traditionally, farmers rely on local traders or intermediaries for price information, which often puts them at a disadvantage due to limited bargaining power and information asymmetry. AI‐powered platforms and mobile applications now collect, process, and analyze market data from different sources and provide farmers with timely updates on commodity prices, demand trends, and market opportunities. Using ML techniques, these systems can also forecast price trends and help farmers decide when and where to sell their produce to maximize returns. By having access to more reliable and timely market information, farmers are better positioned to negotiate prices and avoid exploitative practices by intermediaries [30].

Beyond price information, AI is also helping to improve coordination across agricultural value chains in East Africa. Predictive analytics can be used to anticipate supply and demand patterns, helping stakeholders such as cooperatives, traders, and policymakers make more informed decisions. AI systems can also support logistics planning by identifying efficient transportation routes, reducing delays, and minimizing postharvest losses, which remain a major challenge in the region due to inadequate storage and handling infrastructure. By improving transparency and coordination among different actors in the value chain, AI‐based market intelligence systems contribute to more efficient markets and fairer distribution of value. This not only benefits farmers through better prices but also enhances the overall stability of food systems in the region [9, 10, 13].

Despite these promising developments, the adoption of AI‐driven financial and market intelligence systems in East Africa is still faced with several practical challenges. Limited digital infrastructure, inconsistent internet connectivity, low levels of digital and financial literacy among farmers, and concerns about data privacy can all slow down adoption. In addition, unequal access to smartphones and digital platforms means that not all farmers benefit equally from these innovations. There are also concerns about whether AI models trained on nonlocal or incomplete data can accurately reflect the realities of smallholder farmers in East Africa. Addressing these challenges requires coordinated efforts from governments, private sector actors, financial institutions, and development partners to strengthen digital infrastructure, improve regulatory environments, promote digital literacy, and ensure inclusive and context‐specific AI solutions. With the right support, AI has strong potential to enhance financial inclusion, improve market access, and contribute to more resilient and productive agricultural systems across East Africa [9, 10, 26, 27].

#### 3.1.5. Climate‐Smart Agriculture

In East Africa, climate variability and change continue to pose serious challenges to agricultural production, particularly for smallholder farmers who depend heavily on rain‐fed systems. Increasingly erratic rainfall, frequent droughts, flooding events, and rising temperatures have made farming less predictable and more vulnerable. In this context, AI is playing an important role in supporting climate‐smart agriculture by helping farmers better understand, anticipate, and respond to changing environmental conditions. AI‐powered systems integrate data from satellites, weather stations, and historical climate records to generate localized and timely weather forecasts. These forecasts enable farmers to make more informed decisions regarding planting dates, crop selection, and input application, thereby reducing the risks associated with climate uncertainty and improving resilience [9, 10, 31].

In many parts of East Africa, AI‐driven decision‐support tools are being developed to provide localized and practical recommendations that match specific agroecological conditions. ML models can combine data on soil properties, rainfall patterns, temperature trends, and crop requirements to recommend suitable planting windows, crop varieties, and management practices. These tools are particularly valuable in areas where agricultural extension services are limited or overburdened. Through mobile applications, SMS‐based platforms, and AI chatbots, farmers can receive timely and context‐specific guidance in accessible formats and local languages. This helps bridge the information gap and empowers farmers to adjust their practices in line with seasonal climate variability [13, 32].

AI also strengthens climate risk management by enabling early warning systems for extreme weather events. In East Africa, where droughts and floods frequently affect agricultural livelihoods, AI models use remote sensing data, climate indicators, and predictive analytics to detect early signs of environmental stress. These systems can provide alerts to farmers, extension agents, and policymakers, allowing them to take proactive measures such as adjusting irrigation, delaying planting, or preparing for potential crop losses. Early warning information is particularly critical in reducing the impacts of climate shocks and improving preparedness at both household and institutional levels. In addition, AI‐based climate risk mapping can help identify vulnerable regions and guide targeted adaptation interventions [10, 31, 33].

Another important contribution of AI to climate‐smart agriculture in East Africa is its role in optimizing the use of scarce natural resources such as water and soil nutrients. AI‐enabled precision agriculture tools, including sensor‐based irrigation systems and data‐driven nutrient management platforms, help ensure that inputs are applied efficiently and only when needed. For example, AI can analyze soil moisture data and weather forecasts to determine the optimal timing and amount of irrigation, thereby conserving water while maintaining crop productivity. Similarly, AI models can recommend precise fertilizer application rates based on soil nutrient levels and crop requirements, reducing both input costs and environmental degradation. These technologies support sustainable intensification by improving productivity while minimizing negative environmental impacts [6, 9, 16, 20].

Despite these promising opportunities, the adoption of AI‐driven climate‐smart agriculture in East Africa is still constrained by several challenges. Limited availability of high‐quality localized data, inadequate digital infrastructure, low levels of digital literacy, and restricted access to smartphones and internet services all affect the scalability of AI solutions. In addition, many smallholder farmers continue to rely on indigenous knowledge and traditional forecasting methods, which may not always align with rapidly changing climate patterns. There are also concerns regarding the affordability of AI technologies and the need to ensure that solutions are inclusive and accessible to marginalized groups. Addressing these challenges requires coordinated efforts among governments, research institutions, private sector actors, and development partners to invest in digital infrastructure, strengthen data systems, promote capacity building, and develop farmer‐centered AI solutions. With appropriate support, AI has the potential to significantly enhance climate resilience, improve resource efficiency, and contribute to sustainable agricultural development in East Africa [9, 10, 31, 32].

### 3.2. Adoption of AI Technologies

#### 3.2.1. Adoption Trends (East African Perspective)

The adoption of AI in East African agriculture is gradually increasing, although it remains at an early and uneven stage across countries such as Ethiopia, Kenya, Tanzania, and Uganda. In recent years, governments, development partners, and private sector actors have introduced a range of digital agriculture initiatives that incorporate AI‐driven tools to support agricultural advisory services, market access, and farm management. However, the diffusion of these technologies is still limited to pilot projects and specific user groups rather than being widely embedded in mainstream farming practices. This pattern is consistent with broader observations that digital transformation in African agriculture is progressing but has not yet reached scale among smallholder farmers [1, 34, 35].

Across developing regions, the adoption of digital and AI‐based agricultural advisory services shows both similarities and clear differences. In South Asia (India and Bangladesh), mobile‐based advisory services such as SMS and app‐based systems are widely used by smallholders. However, more advanced AI tools like CV for pest detection are still mostly at pilot stage due to limited digital skills and infrastructure [36, 37].

In West Africa (Nigeria and Ghana), digital agriculture projects are emerging but remain largely experimental. Many initiatives face challenges such as weak rural connectivity, low digital literacy, and limited institutional support for scaling [38].

In Latin America (Brazil and Colombia), precision agriculture is more advanced, including the use of sensors and satellite‐based systems. However, these technologies are mainly used by large and commercial farms rather than smallholders [39, 40].

In East Africa, the key strength is the rapid spread of mobile money systems like M‐Pesa, which supports digital service delivery. This creates a strong foundation for linking financial services with AI‐based agricultural advisory tools. Still, challenges such as limited infrastructure and low digital literacy remain [28, 41].

One of the most prominent trends in the region is the widespread use of mobile‐based platforms as the main channel for delivering AI‐enabled agricultural services. SMS, USSD, and voice‐based systems are particularly important because they are compatible with basic mobile phones, which remain the most accessible digital devices in rural areas. These platforms often integrate AI algorithms to provide farmers with personalized recommendations on weather conditions, pest and disease management, and input use. Evidence suggests that mobile‐based advisory systems significantly enhance farmers′ access to timely and relevant agricultural information, especially in contexts where traditional extension services are limited [8, 11, 42]. In East Africa, such tools have become a practical bridge between advanced digital technologies and low‐resource farming environments.

In terms of geographical distribution, adoption levels vary across East African countries, with Kenya often cited as a regional leader in digital and AI‐driven agricultural innovation. Kenya′s relatively advanced ICT infrastructure, higher mobile money penetration, and active ecosystem of agritech startups have facilitated the development and scaling of AI‐based agricultural platforms. Similar initiatives exist in Ethiopia, Uganda, and Tanzania, but their reach and sustainability are often constrained by infrastructural and institutional limitations. Reports indicate that East Africa is at the forefront of digital agriculture adoption in Africa, yet the benefits are still unevenly distributed among farmers [1, 3, 43, 44]. This suggests that while innovation is growing, scaling remains a key challenge.

Despite the availability of AI‐enabled solutions, adoption remains highly uneven among different categories of farmers. Farmers who have better access to education, extension services, financial resources, and digital literacy are more likely to adopt and effectively use these technologies. On the other hand, smallholder farmers in remote and underserved areas face multiple constraints, including limited awareness of AI tools, lack of reliable internet connectivity, affordability issues, and insufficient training. These barriers are widely documented as major determinants of digital technology adoption in agriculture and continue to shape unequal adoption patterns in East Africa [35, 45]. As a result, AI adoption tends to be concentrated among relatively better‐resourced and more connected farming households.

Institutional and ecosystem factors also play a crucial role in shaping adoption trends in the region. Many AI‐based agricultural interventions in East Africa are introduced through partnerships involving governments, international organizations, NGOs, and private sector firms. These collaborations have contributed to the diffusion of digital advisory services and AI‐powered tools, but they also highlight the dependence on external actors for innovation and scaling. Integration with public extension systems remains limited in many cases, which affects long‐term sustainability and widespread adoption. Studies emphasize that strong institutional frameworks, supportive policies, and coordination among stakeholders are essential for scaling digital agriculture technologies in Africa [1, 9, 43]. Without such support, adoption is likely to remain fragmented and uneven.

Overall, although AI technologies are increasingly recognized as valuable tools for improving agricultural productivity and resilience in East Africa, their adoption is still in a transitional phase. Mobile‐based platforms continue to dominate due to their accessibility, but broader adoption is constrained by structural challenges such as infrastructure gaps, limited digital skills, and institutional weaknesses. Moving forward, enhancing rural connectivity, strengthening extension systems, investing in digital literacy, and fostering locally driven innovation will be critical to accelerating the adoption and impact of AI technologies among smallholder farmers in the region.

#### 3.2.2. Determinants of Adoption

The adoption of AI technologies in East African agriculture is shaped by the everyday realities of smallholder farmers knowledge, access to services, financial capacity, and the broader institutional environment. Although AI tools are increasingly promoted as solutions for improving productivity and resilience, their actual use depends on whether farmers find them accessible, relevant, and practical. Across countries such as Ethiopia, Kenya, Tanzania, and Uganda, several key factors consistently influence adoption decisions.

One of the most important drivers is education and digital literacy. Farmers who have some level of formal education or prior exposure to digital tools are generally more comfortable using AI‐enabled applications, such as mobile advisory platforms or decision‐support systems. In many rural areas, however, limited digital skills remain a major barrier, making it difficult for farmers to fully understand or trust technology‐based recommendations. This highlights the importance of not only general education but also targeted digital skills training. Empirical evidence shows that education and digital literacy significantly increase the likelihood of adopting ICT and AI‐based agricultural innovations [8, 35, 45].

Access to extension services is another critical factor. In the East African context, extension agents are often the bridge between new technologies and farmers. When AI tools are integrated into extension systems such as through mobile‐based advisory services or digital platforms, farmers are more likely to adopt them because they receive guidance from trusted sources. Regular contact with extension workers helps reduce uncertainty, builds confidence, and improves understanding of how to apply new technologies in practice. Studies confirm that participation in extension programs and training significantly increases technology adoption among smallholders [1, 46–48].

The availability of mobile devices and internet connectivity also plays a central role. Because most AI‐based agricultural services in East Africa are delivered through mobile phones, access to these devices is essential. Although mobile penetration has improved significantly in the region, challenges such as limited smartphone ownership, poor network coverage, and high data costs still persist especially in remote rural areas. As a result, simpler technologies like SMS and voice‐based services are more widely adopted than internet‐dependent applications. This digital divide continues to shape who benefits from AI innovations [9, 10].

Economic factors, particularly income level and farm size, strongly influence farmers′ willingness and ability to adopt AI technologies. Farmers with higher incomes or larger landholdings are generally more able to invest in new tools and absorb the risks associated with innovation. In contrast, resource‐constrained smallholders may hesitate to adopt AI solutions due to financial limitations and uncertainty about returns. This creates unequal adoption patterns, where better‐off farmers tend to be early adopters while poorer farmers lag behind. This pattern is widely documented in the agricultural technology adoption literature [49–52].

Finally, institutional support and training are essential for promoting and sustaining adoption. In many East African countries, AI technologies are introduced through government initiatives, NGOs, research institutions, and private sector partnerships. These actors provide training, technical support, and sometimes financial incentives, which help farmers learn how to use new technologies effectively. Without such support, adoption is often slow and difficult to sustain. Evidence shows that farmers who receive training and institutional backing are significantly more likely to adopt and continue using agricultural innovations [1, 9].

Overall, the adoption of AI in East African agriculture is influenced by a combination of human, economic, technological, and institutional factors. For AI technologies to have a meaningful impact, they must be designed with farmers′ realities in mind simple to use, affordable, and supported by strong extension and training systems. Strengthening digital literacy, improving rural connectivity, and enhancing institutional coordination will be key to ensuring that smallholder farmers are not left behind in the digital transformation of agriculture.

#### 3.2.3. Barriers to Adoption (East African Perspective)

Although AI technologies offer promising solutions for improving agricultural productivity and resilience in East Africa, their adoption on the ground remains constrained by several practical challenges. For most smallholder farmers, the issue is not just about the availability of technology but whether it fits within their daily realities, considering limitations in infrastructure, skills, affordability, and data systems. These barriers are deeply interconnected and continue to slow the widespread use of AI in agriculture across the region.

A major constraint is infrastructure limitation. Many rural areas in East Africa still struggle with unreliable electricity and weak internet connectivity. Because most AI‐based agricultural tools depend on digital platforms, cloud systems, or real‐time data access, poor infrastructure makes it difficult for farmers to use them effectively. In remote areas, network coverage can be inconsistent, and power outages are common, which discourages continuous use of digital services. Recent studies emphasize that without strong rural infrastructure, the benefits of digital and AI‐driven agriculture cannot be fully realized [53, 54].

Another important barrier is the digital literacy gap. Although mobile phone access has improved significantly, many farmers still lack the skills needed to use advanced digital tools. For example, navigating smartphone applications, interpreting AI‐generated recommendations, or engaging with digital advisory platforms can be challenging for farmers who have not received adequate training. This limits their confidence and willingness to adopt new technologies. Evidence shows that digital skill gaps remain a major obstacle to the effective use of agricultural innovations in developing regions, including East Africa [55–57].

Cost and accessibility also present serious challenges. The price of smartphones, internet data, and subscription‐based digital services can be too high for many smallholder farmers. Even when pilot projects provide temporary access to these technologies, sustaining their use becomes difficult once external support ends. As a result, poorer farmers are often excluded, whereas relatively better‐off farmers are more able to adopt and benefit from AI tools. Studies highlight that affordability remains one of the most critical barriers to scaling digital agriculture solutions in low‐income rural settings [54, 58].

In addition, data‐related challenges limit the effectiveness of AI technologies in agriculture. AI systems rely heavily on accurate, timely, and location‐specific data to generate useful recommendations. However, in many parts of East Africa, agricultural data systems are still underdeveloped. There is often a lack of reliable information on weather patterns, soil conditions, crop performance, and market trends. This can reduce the accuracy of AI‐based advice and weaken farmers′ trust in these systems. Strengthening data collection, management, and sharing mechanisms is therefore essential for improving the reliability and impact of AI in agriculture [10, 59, 60].

Overall, athough AI technologies have strong potential to transform agriculture in East Africa, these barriers highlight the gap between innovation and practical use. Addressing infrastructure challenges, improving digital skills, making technologies more affordable, and strengthening data systems will be key to ensuring that AI becomes a meaningful and accessible tool for smallholder farmers.

### 3.3. Implications for Smallholder Farmers

The introduction of AI technologies into agriculture is beginning to reshape how smallholder farmers in East Africa access information, make decisions, and manage their farms. Although the impacts are still emerging, there is growing evidence that AI can bring meaningful benefits, but also new risks that need careful attention. Understanding these implications is essential for ensuring that AI contributes positively to rural livelihoods.

#### 3.3.1. Productivity and Efficiency

AI technologies are helping farmers make more informed and timely decisions, which can lead to improved productivity and efficiency. Through mobile‐based advisory platforms, farmers can receive recommendations on planting time, fertilizer application, pest control, and weather forecasts tailored to their local conditions. This allows them to use inputs more efficiently and avoid unnecessary costs.

In parts of East Africa, farmers using digital and AI‐supported advisory services have reported better crop management practices, increased yields, and reduced input waste. For example, precision recommendations on fertilizer use or pest control help farmers avoid overuse while maintaining productivity. Studies show that digital advisory tools can significantly improve farm outcomes by enhancing decision‐making and reducing uncertainty [1, 8, 9, 61]. In this sense, AI is not replacing farmers′ knowledge but strengthening it with timely and data‐driven insights.

#### 3.3.2. Improved Access to Information

One of the most immediate benefits of AI in East African agriculture is improved access to information. In many rural areas, the number of extension agents is limited, and farmers often struggle to get timely advice. AI‐powered tools, especially those delivered through mobile phones, help bridge this gap by providing real‐time, location‐specific information directly to farmers.

These systems can deliver weather updates, market prices, and agronomic advice in local languages, making them more accessible to a wide range of users. This is particularly important in remote areas where traditional extension services are weak or overstretched. Evidence suggests that digital platforms can complement extension systems and significantly expand the reach of agricultural information services [10, 57, 62]. As a result, farmers are better equipped to respond to changing conditions and make informed decisions.

#### 3.3.3. Inclusivity and Gender Considerations

AI technologies also have the potential to promote inclusivity in agriculture, particularly for women and other marginalized groups who often face barriers in accessing information and resources. When designed thoughtfully, digital tools can reach farmers who are traditionally excluded from extension services due to social, cultural, or geographical constraints.

For example, mobile‐based advisory services can provide women farmers with direct access to agricultural knowledge without requiring travel or formal institutional engagement. This can help reduce information gaps and improve their participation in agricultural decision‐making. However, inclusivity is not automatic, it depends on how technologies are designed and implemented. Studies emphasize that gender‐sensitive approaches, local language support, and affordable access are essential for ensuring that AI benefits all farmers equally [58, 59, 63, 64].

It is important to recognize that access to digital technology is uneven across social groups, and this shapes who benefits from AI‐based agricultural innovations. In East Africa, women are significantly less likely to own mobile phones especially smartphones compared to men, with gender gaps in mobile ownership estimated at around 15%–20% [65]. Beyond ownership, women also face barriers such as lower levels of digital literacy, limited control over income, and sociocultural norms that restrict access to information and technology [41].

Similar exclusion patterns are observed among farmers in remote rural areas, pastoralist communities, and young people with limited access to land and resources. These groups often lack reliable internet connectivity, formal training opportunities, and affordable digital services, which reduces their ability to use AI‐driven advisory tools effectively [38].

Therefore, without deliberate and inclusive interventions—such as subsidized smartphones, women‐targeted digital literacy programs, and community‐based digital access centers—there is a real risk that AI technologies could reinforce or even deepen existing inequalities rather than reduce them [41, 65].

#### 3.3.4. Risks and Concerns

Despite its potential, the use of AI in agriculture also raises several concerns that need to be addressed.

• Over‐reliance on AI systems may gradually reduce the value placed on indigenous knowledge and traditional farming practices, which have been developed over generations and are often well adapted to local conditions. Balancing modern technology with local knowledge is therefore important.
• Potential job displacement is another concern, particularly as automation and digital tools begin to replace certain labor‐intensive tasks. Although this may increase efficiency, it could also reduce employment opportunities in rural areas if not managed carefully.
• There is also a risk of widening inequality. Farmers who have access to digital tools, education, and financial resources are more likely to benefit from AI, whereas poorer and less connected farmers may be left behind. This digital divide could deepen existing socioeconomic inequalities if inclusive strategies are not prioritized [54, 55, 60].

Overall, AI technologies offer significant opportunities to improve productivity, access to information, and inclusivity for smallholder farmers in East Africa. However, these benefits are not guaranteed. To ensure positive outcomes, there is a need for inclusive policies, strong extension support, investment in digital infrastructure, and careful integration of local knowledge systems.

### 3.4. Policy and Development Implications

To fully realize the potential of AI in East African agriculture, supportive policies and coordinated development efforts are essential. Although AI technologies are increasingly available, their impact will largely depend on how well governments, development partners, and private sector actors work together to create an enabling environment. The following strategies are critical for scaling AI adoption among smallholder farmers in the region.

One of the most important priorities is investment in rural infrastructure, particularly in improving access to electricity and reliable internet connectivity. Many rural communities in East Africa still face challenges related to power supply and network coverage, which limits the use of digital and AI‐based tools. Expanding rural electrification and broadband connectivity can significantly enhance farmers′ ability to access mobile‐based advisory services, digital platforms, and real‐time agricultural information. Infrastructure development is widely recognized as a foundational requirement for digital agriculture transformation [53, 54].

Another key area is capacity building and digital literacy programs. Even when technologies are available, farmers need the skills to use them effectively. Training programs that focus on digital skills, basic ICT use, and understanding AI‐driven recommendations can empower farmers to confidently engage with digital tools. These programs should be tailored to rural contexts and delivered through extension systems, farmer organizations, and community‐based initiatives. Evidence suggests that building digital capacity among farmers significantly increases the likelihood of adopting and benefiting from agricultural technologies [55–57].

Strengthening agricultural extension systems is also crucial. Extension agents play a central role in linking farmers with new technologies, including AI‐based tools. By equipping extension workers with digital skills and integrating AI platforms into extension services, governments can enhance the reach and effectiveness of agricultural advisory systems. Hybrid models that combine human extension with digital advisory services have been shown to improve information dissemination and farmer uptake of innovations [1, 9].

The promotion of public–private partnerships (PPPs) is another important strategy. Many AI innovations in agriculture are developed and delivered by private sector actors, including agritech startups and mobile service providers. Governments can collaborate with these actors to scale solutions, improve service delivery, and ensure affordability. PPPs can also help align technological innovation with national agricultural priorities and sustainability goals. Such partnerships have been identified as key drivers of digital transformation in agriculture across developing regions [3, 10].

Finally, there is a need to focus on developing inclusive and context‐specific AI solutions. AI technologies must be designed to reflect the realities of East African smallholder farmers, including language diversity, limited connectivity, low literacy levels, and varying socioeconomic conditions. Inclusive design ensures that technologies are accessible to women, youth, and marginalized groups who often face additional barriers to access. Localization of content, affordability, and user‐friendly interfaces are essential for ensuring that AI solutions are widely adopted and effective. Research highlights that context‐specific and inclusive digital solutions are more likely to achieve meaningful impact in smallholder farming systems [58–60].

In summary, accelerating AI adoption in East African agriculture requires a holistic approach that goes beyond technology provision. It involves strengthening infrastructure, building human capacity, enhancing institutional systems, fostering collaboration among stakeholders, and ensuring that innovations are inclusive and tailored to local needs. With the right policy support and coordinated efforts, AI has the potential to significantly contribute to agricultural transformation and rural development in the region.

### 3.5. Research Gaps and Future Directions

Despite the growing interest in AI in agriculture, several important research gaps remain, particularly in the East African context. Addressing these gaps is essential for ensuring that AI technologies are effectively designed, implemented, and scaled for smallholder farmers.

One key gap is the limited evidence on long‐term impacts of AI technologies. Most existing studies focus on short‐term outcomes such as improved access to information or initial productivity gains, with less attention given to sustainability, long‐term adoption, and enduring impacts on livelihoods.

Another important gap is the insufficient focus on gender and social inclusion. Many AI‐based agricultural solutions do not fully consider the specific needs and constraints of women, youth, and marginalized groups, which may limit equitable access and benefits.

There is also a lack of localized and high‐quality datasets for training AI systems. Without reliable, context‐specific data, AI tools may produce less accurate or less relevant recommendations for farmers in different agroecological zones.

In addition, there is a need for more interdisciplinary research that combines technical, social, and economic perspectives. Many studies tend to focus either on the technological aspects or on adoption behavior, rather than integrating both dimensions to better understand real‐world application.

Future research should therefore emphasize participatory and farmer‐centered approaches, where farmers are actively involved in the design, testing, and evaluation of AI technologies. This will help ensure that innovations are practical, inclusive, and aligned with the actual needs of smallholder farming systems.

### 3.6. Summary Tables and Analytical Figures

To better organize and interpret the evidence reviewed in this study, this section brings together key findings using summary tables and figures. These help to clearly show where AI in agriculture is being studied most in East Africa, what drives or limits adoption, and what gaps still exist in the literature [39, 66].

The distribution of studies shows clear differences across countries. Kenya leads in almost all AI application areas, which reflects its stronger digital agriculture ecosystem and active innovation landscape. Ethiopia also shows a growing body of research, driven by increasing government attention to digital agriculture and extension reforms (Table 1).

**Table 1:** Distribution of included studies by East African Country and AI Application Area.

Country	AI advisory	Precision agriculture	Crop/pest monitoring	Financial/market intelligence	Climate‐smart	Total
Kenya	12	8	10	15	7	52
Ethiopia	8	5	6	4	6	29
Tanzania	6	3	4	5	4	22
Uganda	5	2	3	3	3	16
Rwanda	2	1	1	2	2	8
Regional (multicountry)	4	2	3	4	3	16
Total	37	21	27	33	25	143∗

*Note:* Sources are from different literatures.

Most AI applications focus on advisory services and market intelligence tools, showing that digital agriculture in East Africa is currently more focused on improving decision‐making and market access rather than only production technologies [66, 67].

Table 2 summarizes what helps farmers adopt AI technologies and what holds them back. Overall, the same challenges appear across most East African countries, especially related to skills, infrastructure, and affordability.

**Table 2:** Key determinants and barriers to AI adoption among smallholder farmers.

Category	Factors supporting adoption	Barriers limiting adoption	Strength of evidence
Human capital	Education, digital literacy	Low ICT skills, limited awareness	Strong
Socioeconomic	Higher income, larger farms	Poverty, high cost of devices/data	Strong
Infrastructure	Mobile coverage, electricity	Poor connectivity, power shortages	Strong
Institutional	Extension services, training	Weak integration, low trust	Moderate
Technological	Simple design, local language tools	Poor localization, weak models	Moderate
Data systems	—	Lack of data systems, privacy concerns	Emerging

*Note:* Sources are from different literatures.

The most serious barriers are not only technological, but also structural especially low digital skills and weak rural infrastructure. Without addressing these, even well‐designed AI tools may have limited impact [39, 68].

Table 3 above reveals noticeable differences in the adoption of AI‐enabled agricultural services across East African countries. Kenya leads in AI utilization among smallholder farmers, whereas Ethiopia shows the lowest adoption level, suggesting that access to smartphones and digital agricultural services plays an important role in promoting the use of AI technologies in agriculture. Although research on AI in East African agriculture is growing, several important gaps remain. Most studies are still short‐term and pilot‐based, which limits understanding of long‐term impacts (Table 4).

**Table 3:** Comparative adoption of AI‐enabled agricultural services in East Africa (2024 estimates).

Country	Smartphone ownership (rural)	% smallholders using any digital ag service	% using AI‐powered service	Leading AI service type
Kenya	45%	28%	12%	Credit scoring, advisory
Ethiopia	22%	12%	4%	Pest detection
Tanzania	30%	18%	6%	Market prices
Uganda	28%	15%	5%	Weather advisory
Rwanda	35%	20%	7%	Soil mapping

**Table 4:** Research gaps and future directions.

Research gap	Priority	Future research direction
Long‐term impacts	High	Conduct longitudinal studies on income, productivity, and welfare over time
Gender and inclusion	High	Focus on women farmers and vulnerable groups using disaggregated data
Local AI models	High	Develop context‐specific, low‐cost AI and data systems
Interdisciplinary research	Medium	Combine technical, social, and economic perspectives
Scalability	Medium	Study what makes AI solutions succeed or fail at scale

Future research needs to move beyond pilot projects and focus more on long‐term, inclusive, and scalable solutions that reflect real farming conditions in East Africa [66].

#### 3.6.1. Conceptual Framework for AI Adoption in East African Agriculture

**Figure 2:**
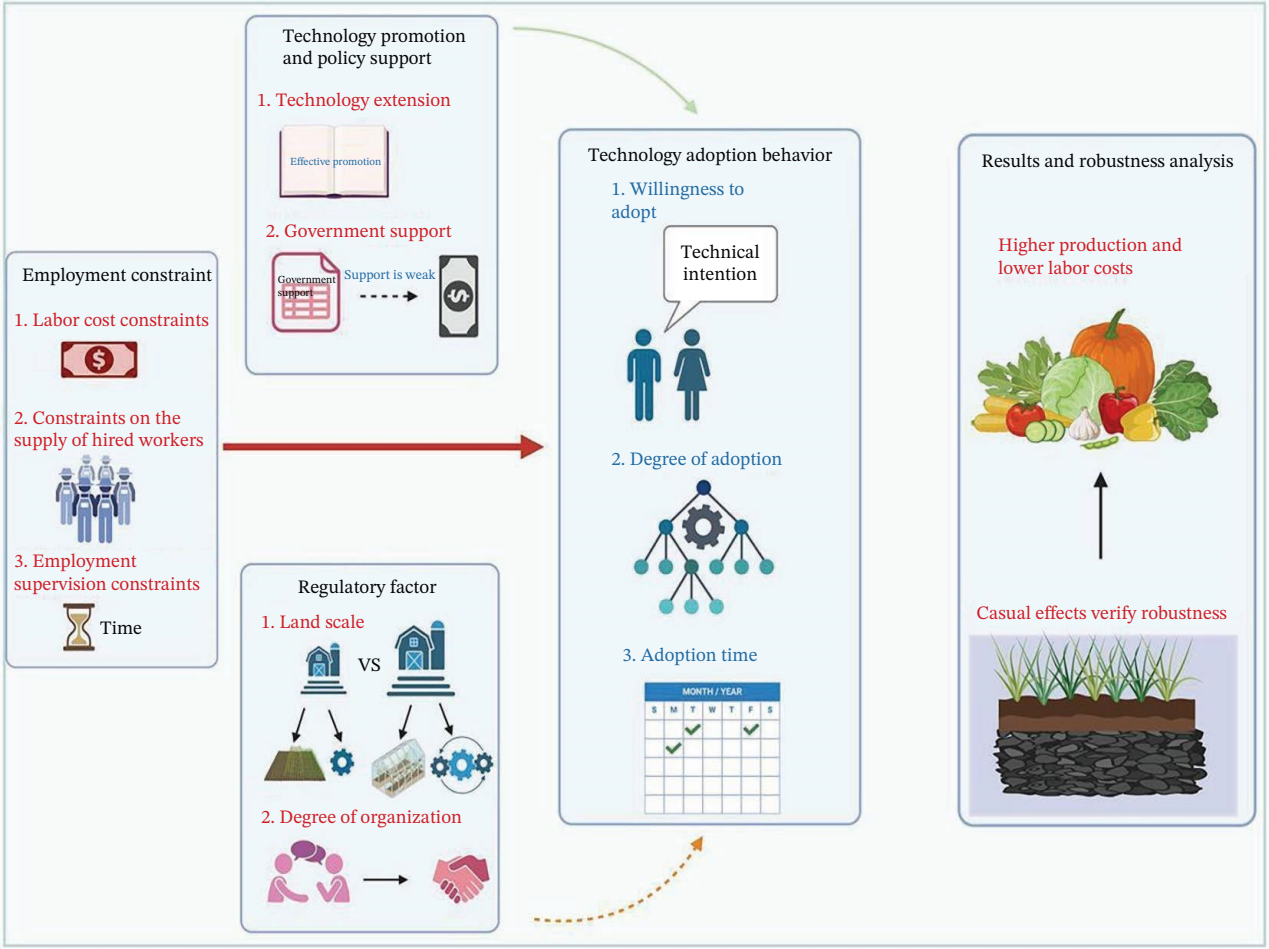
Conceptual Framework for AI Adoption in East African Agriculture.

This framework explains AI adoption in agriculture through three simple layers. First, farmers operate in challenging contexts marked by climate risks, small land sizes, and weak infrastructure. Second, adoption depends on how affordable, simple, and locally relevant the AI tools are. Third, individual and system factors such as education, income, infrastructure, and institutions determine whether farmers actually use these tools. When adoption happens, it improves productivity, information access, and resource use efficiency, which in turn builds income and confidence and encourages further adoption over time (Figure 2).

**Figure 3:**
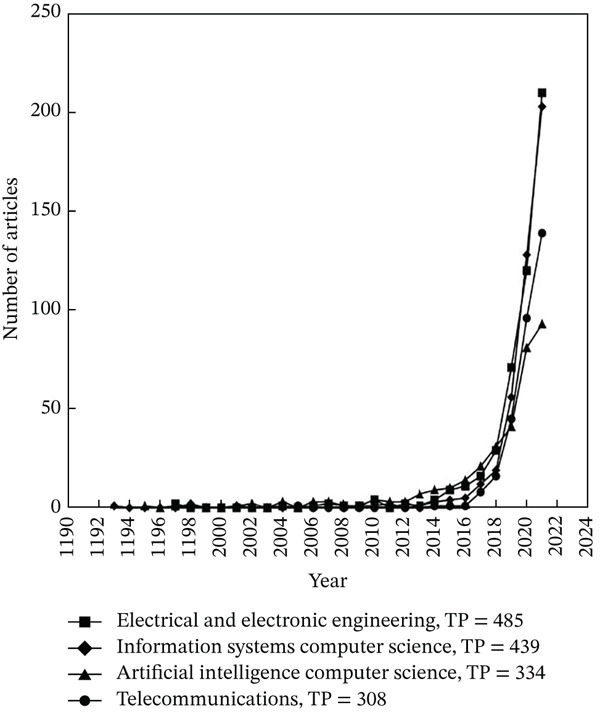
Publication Trend of AI in Agriculture Research in East Africa (1985–2025).

Before 2015, research on AI in agriculture was still very limited. However, things changed quickly after 2015, with a sharp rise in publications. This growth has been driven by several practical developments, including the spread of mobile‐based advisory services, wider use of precision farming tools, increased donor support for digital agriculture initiatives, and growing interest from researchers in AI and ML for farming (Figure 3). Overall, AI in agriculture is still an emerging field in East Africa, but it is growing quickly and gaining strong attention [39, 67].

## 4. Conclusion and Recommendation

This review shows that AI has real potential to support agricultural development in East Africa, especially for smallholder farmers who continue to face challenges related to limited access to timely information, low productivity, and increasing climate risks. AI‐based tools, particularly those delivered through mobile phones, are gradually helping farmers make better decisions, access advisory services, and manage their farms more efficiently. In this sense, AI is emerging as a useful support tool rather than a replacement for farmers′ own knowledge and experience.

At the same time, the review makes it clear that the adoption of AI technologies is still constrained by several practical challenges. Many rural communities lack reliable infrastructure such as electricity and internet connectivity, whereas others face barriers related to cost, limited digital skills, and inadequate institutional support. These challenges make it difficult for many smallholder farmers to access and effectively use AI‐based solutions, meaning that the benefits of these technologies are not yet evenly distributed across the region.

The review also emphasizes that AI adoption is closely linked to broader development conditions. Factors such as education, access to extension services, availability of mobile devices, and supportive policies all influence whether farmers are able to engage with and benefit from AI technologies. This highlights the importance of viewing AI not only as a technological innovation but as part of a wider agricultural and rural development system.

In addition, the study draws attention to the importance of inclusivity. Without intentional efforts to address issues such as gender inequality and the digital divide, there is a risk that AI technologies may primarily benefit better‐resourced farmers while leaving more vulnerable groups behind. Ensuring that AI solutions are designed in a way that is accessible, affordable, and relevant to diverse farming communities is therefore essential.

Overall, although AI presents promising opportunities for improving agriculture in East Africa, its impact will depend on how well it is supported by infrastructure, institutions, and inclusive policies. A farmer‐centered approach that combines technological innovation with capacity building, strong extension systems, and equitable access will be key to achieving sustainable and meaningful improvements in smallholder farming systems.

To enhance the adoption and impact of AI in East African agriculture, several practical steps are needed.

First, improving rural infrastructure—especially electricity and internet access—is essential, as these are the foundation for using most AI‐based tools. Without them, access will remain limited for many smallholder farmers.

Second, building farmers′ digital skills through training and awareness programs is important. Strengthening the capacity of extension workers to support farmers in using AI tools can also improve adoption and effective use.

Third, integrating AI into agricultural extension services can help deliver timely and relevant advice to farmers, combining digital tools with human support for better results.

Fourth, encouraging collaboration between governments, private sector actors, and development partners can support the development and scaling of affordable, farmer‐friendly AI solutions.

Finally, AI tools should be designed to be inclusive and context‐specific, taking into account the needs of women, youth, and marginalized farmers, as well as local farming conditions and languages.

Overall, a combined effort that focuses on infrastructure, skills, inclusivity, and collaboration will help ensure that AI technologies truly benefit smallholder farmers in the region.

## Author Contributions

Zebenay Shitaye led the overall work, from developing the research idea, and reviewing the literature to analyzing the findings and writing the first draft of the manuscript. Manale Andargie Embyale contributed to shaping the study design and provided valuable feedback that helped improve the structure and quality of the paper. Tewodros Adane Nega supported the interpretation of results and helped strengthen the methodological aspects of the study. Getachew Eshetu Gidelew contributed by carefully reviewing the manuscript and enriching the discussion with important insights. Marta Mohammed played a key role in editing and refining the manuscript, ensuring clarity and coherence throughout.

## Funding

No funding was received for this research.

## Disclosure

All authors have read and approved the final version of the manuscript.

## Ethics Statement

This study is based on a systematic review of existing literature and does not involve human participants or animals; therefore, ethical approval was not required.

## Consent

As the study does not involve human participants, consent to participate is not applicable.

## Conflicts of Interest

The authors declare no conflicts of interest.

## Data Availability

The data that support the findings of this study are available from the corresponding author upon reasonable request.
